# Changes in Levels of Seminal Nitric Oxide Synthase, Macrophage Migration Inhibitory Factor, Sperm DNA Integrity and Caspase-3 in Fertile Men after Scrotal Heat Stress

**DOI:** 10.1371/journal.pone.0141320

**Published:** 2015-10-29

**Authors:** Mei-Hua Zhang, Ai-Dong Zhang, Zhi-Da Shi, Lei-Guang Wang, Yi Qiu

**Affiliations:** Key Laboratory of Birth Regulation and Control Technology of National Health and Family Planning Commission of China, Key Laboratory for Improving Birth Outcome Technique, Shandong Provincial Family Planning Institute of Science and Technology, 69 Yuhan Road, Jinan, Shandong, 250002, China; University Hospital of Münster, GERMANY

## Abstract

**Background:**

This study observes changes in levels of seminal nitric oxide (NO), nitric oxide synthase (NOS), macrophage migration inhibitory factor (MIF), sperm DNA integrity, chromatin condensation and Caspase-3in adult healthy men after scrotal heat stress (SHS).

**Methods:**

Exposure of the scrotum of 25 healthy male volunteers locally at 40–43°C SHS belt warming 40 min each day for successive 2 d per week. The course of SHS was continuously 3 months. Routine semen analysis, hypo-osmotic swelling (HOS) test, Aniline blue (AB) staining, HOS/AB and terminal deoxynucleotidyl transferase-mediated d UDP nick-end labeling (TUNEL) were carried out before, during and after SHS. Seminal NO and NOS contents were determined by nitrate reduction method. The activated Caspase-3 levels of spermatozoa and MIF in seminal plasma were measured by the enzyme-linked immunosorbent assay (ELISA) method. Statistical significance between mean values was determined using statistical ANOVA tests.

**Results:**

The mean parameters of sperm concentration, motile and progressive motile sperm and normal morphological sperm were significantly decreased in groups during SHS 1, 2 and 3 months compared with those in groups of pre-SHS (P<0.001). Statistically significant differences of sperm DNA fragmentation, normal sperm membrane, and Caspase-3 activity as well as the level of NO, NOS and MIF in semen were observed between the groups before SHS and after SHS 3 months and the groups during SHS 1, 2 and 3 months (P<0.001). After three months of the SHS, various parameters recovered to the level before SHS. WBC in semen showed a positively significant correlation with the levels of NO, NOS, MIF and Caspase-3 activity. The percentage of abnormal sperm by using the test of HOS showed a positively significant correlation with that of HOS/AB.

**Conclusions:**

The continuously constant SHS can impact the semen quality and sperm DNA and chromatin, which may be contributed to the high level of NO, NOS, MIF and Caspase-3 by SHS.

## Introduction

In most mammals, male germ cells should be maintained below body temperature for proper development. Transient scrotal heat stress (SHS) or transient scrotal hyperthermia may cause alteration of sperm parameters and testicular germ cell apoptosis [1–4]. Previous studies on azoospermia or oligozoospermia induced by SHS mainly focused on germ cell apoptosis [5–7]; no data regarding their possible effect on nitric oxide (NO), nitric oxide synthase (NOS) and macrophage migration inhibitory factor (MIF) in semen are available. NO, NOS and MIF are important regulators in many physiologic processes. NO is synthesized through the enzymatic conversion of L-arginine to L-citrulline by the action of one of the isoenzymes known as nitric oxide synthase (NOS), and is concerned with physiological functions in the human male reproductive tracts [8,9]. Low concentrations of NO are necessary to complete a group of male reproductive functions such as spermatogenesis, spermiogenesis, sperm motion, acrosome reaction, sperm/oocyte fusion and sperm capacitation [10], however, high concentrations of NO have injurious effects on sperm properties such as motility, morphology and DNA stability, and to increase apoptosis and block all sperm functions [11–13]. MIF is a T-cell cytokine and present in large quantities in human semen, and is one of the proteins transferred to spermatozoa during the epididymal transit [14,15]. MIF secreted in the epididymis and correlated with sperm maturation and stability [14,15]. However, it is unknown how MIF influences sperm function and apoptosis after scrotal heat stress. Up to the present, the effect of NO, NOS and MIF on human sperm concentration, motility, morphology and DNA stability has not been elucidated clearly.

The hypo-osmotic swelling (HOS) test and the Eosin Y (EY) staining can evaluate sperm alive or dead and detect the integrity of the tail membrane of sperm [16–18]. The terminal deoxynucleotidyl transferase-mediated d UDP nick-end labeling (TUNEL) [19], single cell gel electrophoresis (COMET) assay [20], the sperm chromatin dispersion (SCD) test, and aniline blue (AB) staining [21] are all simple, less expensive procedures and can be performed in a short period of time, and chromatin condensation is vital for the function of the spermatozoon as the motile carrier of the paternal genome. However, the vitality and the integrity of the same sperm membrane could not be determined using these tests [16–19].

Caspase (Cysteine-requiring Aspartate Protease) is a protease family, which plays an important role in apoptosis process. Caspase-3 is a key enzyme in apoptosis process. Of all the Caspases, Caspase-3 is the most studied in mammalian cells. Activated Caspase-3 and loss of the integrity of the DNA fragmentation are other markers of terminal apoptosis expressed by a varying proportion of ejaculated sperm [22–24]. It has been hypothesized that sperm cell death is associated with male infertility. However, the exact mechanisms of its involvement and elevated intra-testicular temperature remain to be elucidated.

The objective of this study was to evaluate whether semen parameters, seminal NO, NOS and MIF, and sperm DNA integrity, chromatin condensation and activity of sperm Caspase-3 are altered in 25 adult healthy men after scrotal heat stress (SHS). On the other hand, the correlations between the levels of NO, NOS, MIF in seminal plasma and methods of determining sperm DNA integrity, sperm chromatin condensation and Caspase-3 were explored.

## Materials and Methods

### Development of Scrotal Heat Stress Device (SHSD)

**Fig 1:**
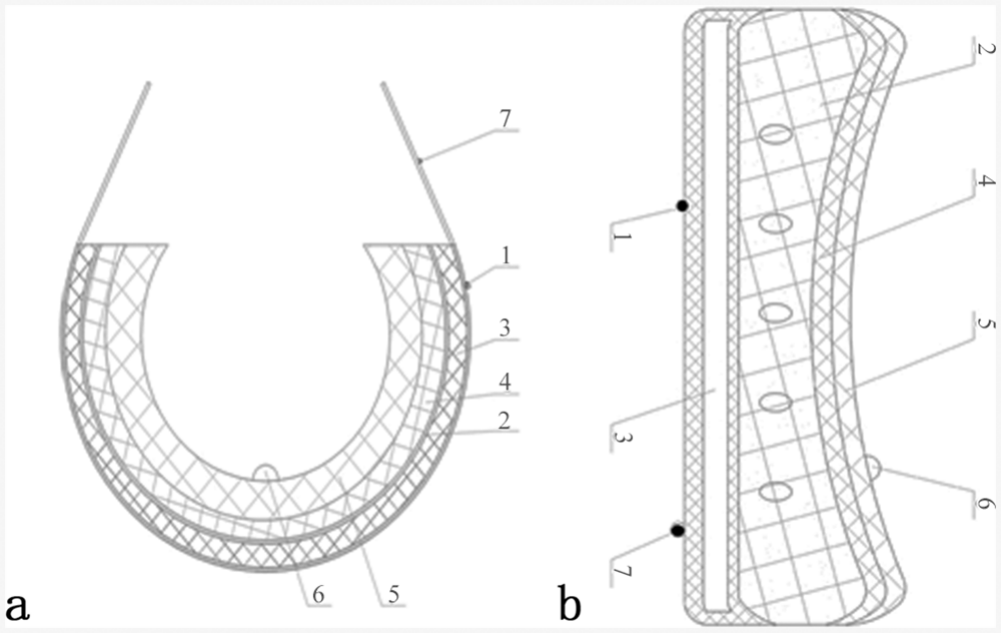
Designed sketch of SHSD. No. 1 and 7 show the fixed belts, No.2 shows the water bag, No. 3 shows the electric heating pad, No. 4 shows the protective layer of the water bag, No. 5 shows the thermal insulation layer and No. 6 shows the temperature probe which links with a digital display. (a) vertical section map of SHSD, (b) cross-section map of SHSD.

SHSD involved water bag, electric heating system, timer and temperature controller, and temperature probe (Fig 1). The water bag was attached to the underpants by 4 belts. The input and output Voltage of the SHSD’s electric heating system were 200 V and 12 V, respectively. The time was 1–60 min. The temperature ranges were from 40.0°C to 43.0°C.

### Equipment and Reagents

Computer-aided sperm analysis (CASA) machines (WLJY-9000, Weili, Beijing, and Fangzheng Beijing University, China) were used for kinematic analysis of spermatozoa. A Leica microscopy (DM4000B; Leica, Wetzlar, Germany) and a phase-contrast microscopy (IX51, Olympus, Japan) were used to observe sperm and cells. Ultraviolet and visible spectrophotometer (TU-1810, Beijing Perkinje General Instrument Co., LTD, PGENERAL) was determined for seminal nitric oxide (NO) and nitric oxide synthase (NOS). The Caspase-3 activity was determined by the Microtiter Plate Reader (ELISA-reader, SMP500-18410-EMKX Versa Max, Molecular Devices Corporation, USA). Sperm Morphology Staining Kit (Diff Quik, YZB-0058-2011) and Aniline Blue Kit were purchased from Hua Kang (YZB-0058-2012, Hua Kang Co. LTD. Shenzhen, China). The TUNEL In-Situ Cell Death Detection Kit, Nitric Oxide (NO) Kit, Nitric Oxide Synthase (NOS) Kit and Caspase-3 Activity Assay Kit were purchased from Bioengineering Institute of Nanjing Jiancheng (Nanjing, Jiangsu, China).

### Volunteers

Between February 2012 and November 2014, twenty-five healthy adult male volunteers, who already had previously fathered at least one child, were recruited into our study. The mean age of them was 34.6 ± 4.5 years (range, 27–43 years). Prior to this study, subjects were informed of the investigations and provided consent. This study has been approved by the Institutional Review Board (IRB) and reviewed by the Ethics Committee of Shandong Provincial Institute of Science and Technology for Family Planning in China. Each of the 25 subjects had provided semen samples for this study. The approval of the institutional research ethics committee and signed written consent of every subject included in the study were obtained. A detailed medical history was taken and physical examination was performed. Subjects currently on any medication or antioxidant supplementation were not included. Physical examination of subjects before, during and after the SHS included the secondary sexual character, scrotum, penis, spermatic cord, vas deferens, testis and epididymis. In addition, subjects were also asked about their sex life and with or without erectile dysfunction, anejaculation or other symptoms. The identification information of each subject in this study was kept confidential and was protected from the public.

Volunteers were chosen for the experiments of wearing 40–43°C SHS, two days per week, and 40 min per day, for a period of 3 consecutive months. Semen was collected at 2 and 1 weeks before, and 1, 2 and 3 months during heat treatment and 1, 2 and 3 months after the last SHS.

### Routine Semen Analysis

Semen samples from the 25 healthy subjects were collected by masturbation and ejaculated into sterile glass cups after 3 to 5 days of abstinence before being were analyzed on-site for both macroscopic and microscopic characteristics within one hour of collection. Information regarding the semen source was withheld from the technologists who performed the semen analysis. Sperm count, percentage of total and progressive motility sperm were determined by using a CASA system. Management of semen aliquots and CASA system operation was realized by a specialized technician experienced in the handling of semen. At least 500 sperm were evaluated per sample (∼8–10 fields), and means for each of the motility parameters [total motility (grade a + b +c %), progressive motility (a +b %)] and sperm concentration were recorded. Density count of leukocytes in seminal plasma was referred to peroxidase dyeing method, recommended by the World Health Organization (WHO) criteria. Sperm morphology was assessed using the strict (WHO) after Diff-Quik staining [25,26].

### Hypo-osmotic Swelling (HOS) Test

HOS test was performed to determine sperm vitality and as previously reported [16–18].

### TUNEL Assay

The TUNEL assay was performed using the In-Situ Cell Death Detection Kit. The fixed slides were rinsed in PBS, pH 7.4, and then permeated with 2% Triton X-100. The terminal deoxynucleotidyl transferase-labeled nucleotide mixture was added to each slide, and these were incubated in a humidified chamber at 37°C for 60 min in the dark. Next, the slides were rinsed 3 times in PBS, with each rinse lasting 5 min, and then converter peroxidase solution was added to the samples. The slides were incubated in a humidified chamber for 30 min at 37°C, rinsed 3 times in PBS, and were then incubated in the presence of diaminobenzidine substrate for 10 min at 15°C to 25°C. The slides were further rinsed 3 times with PBS and counterstained with 2% methyl green. A total of 500 sperm per patient were assessed by the same examiner using bright-field microscopy. First, the total number of sperm per field that were stained with methyl green was counted. Next, the number of brown cells (TUNEL positive) was then counted, and this number was expressed as the percentage of the total sperm cells. Negative (omitting the enzyme terminal transferase) and positive (incubation with deoxyribonuclease I, 1 U/mL for 20 min at room temperature) controls were performed for each experiment. The final percentage of sperm with fragmented DNA was referred to as the percentage of TUNEL-positive sperm.

### Aniline Blue (AB) Staining and HOS (HOS/AB)

After the HOS test had been performed, the AB test was carried out immediately to evaluate the sperm chromatin condensation of the 25 subjects. A total of 0.5 mL of the semen sample (mixed with hypoosmotic medium) from the HOS test was centrifuged at 500 g for 5 min, and then the supernatant was discarded and the sperm pellet was resuspended in 0.1 mL of a hypo-osmotic solution. To perform AB staining, sperms were stained with AB as described in a previous report [27,28]. The slides were prepared by smearing 10 μL of each HOS semen sample. The slides were air-dried and then fixed with a solution of methanol (Hua Kang Co. LTD. Shenzhen, China) in 0.2M phosphate buffer (pH = 7.2) for 1.5 min at room temperature. Slides were then stained with 5% aqueous aniline blue solution mixed with 4% acetic acid (pH = 3.5) for 5 min. This was followed by rinsing and air drying of the slides. For each stained smear, 200 spermatozoa were evaluated with light microscope in oil immersion magnification (100 × objective) (DM4000B; Leica, Wetzlar, Germany). Spermatozoa with unstained (no or light aniline blue-stained) nuclei are considered as normal (clear, mature chromatin) while those blue stained (intermediate and dark aniline blue-stained) were abnormal (immature chromatin) (Fig 1). Results were expressed as the percentage of nuclear unstained and stained sperm. An ejaculate with a rate of blue-stained nucleus sperm less than 20% was considered normal.

**Fig 2:**
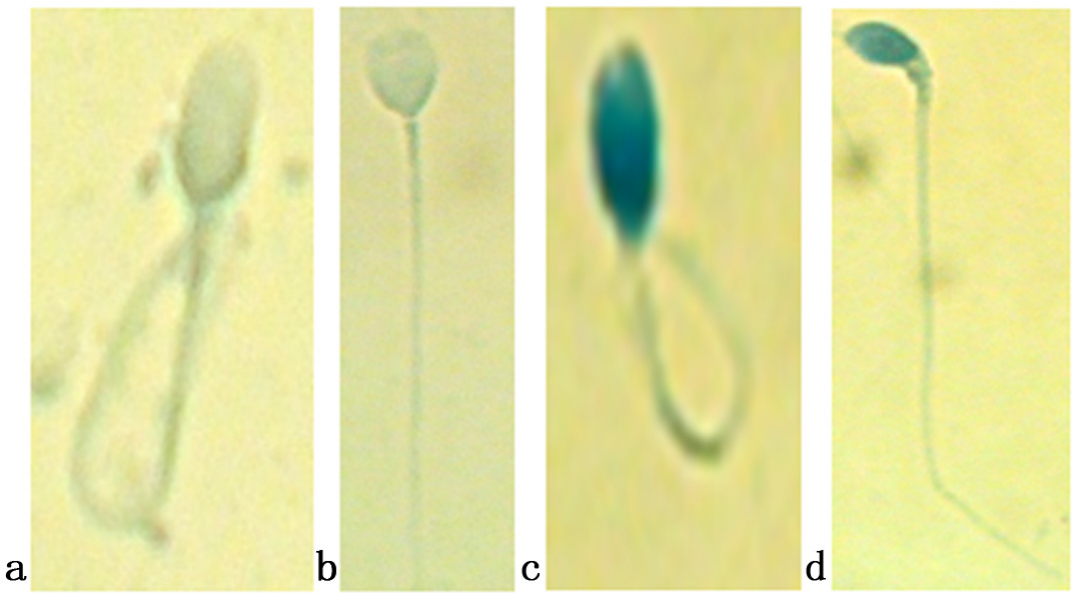
The hypo-osmotic swelling test and aniline blue (AB) staining (HOS/AB) of the same sperm. Patterns observed for the HOS/AB test: (a) AB1, sperm unstained and swollen tails; (b) AB2, sperm unstained and unswollen; (c) AB3, sperm darkly stained and swollen tails; (d) AB4, sperm darkly stained and unswollen.

The patterns observed for the HOS/AB test were classified as follows (Fig 2): AB1, mature sperm, having completed histone transition protein–protamine replacement, stained lightly or unstained and swollen tails, membrane intact and no chromatin defects; AB2, spermatozoa with lightly or unstained head and unswollen tails (membrane damaged and no chromatin defects); AB3, spermatozoa with intermediate or dark aniline blue-stained head and swollen tails (chromatin defects and membrane intact); and AB4, spermatozoa with intermediate or dark aniline blue-stained head and unswollen tails (chromatin defects and membrane damaged, severely immature sperm).

### Caspase-3 Activity

Caspase-3 activity was detected with assay kit of the manufacturer's protocol (Nanjing Jiancheng, China). About 3–5×10^6^ sperms from the semen samples were collected. After centrifugation (2 000 rpm, 5 min), the supernatant was carefully removed, making sure that no cells are removed by suction, the pellet was washed with 300 μl PBS once. The previous process was repeated once before adding lysis buffer to the pellet according to the ratio of 50 μl lysis buffer/2 million cells. Pellet was resuspended and cleaved 30 min in the ice bath with shaking 3~4 times, 10 sec each time, or freezing and thawing 2–3 times. Then the sperm samples were centrifuged for 10–15 min at 4°C (12 000 rpm). The supernatant (containing protein cleavage) was transferred to fresh tubes and put on ice and added 0.5 μl DTT to per 50 μl lysate, incubated for 1 h. Then 5 μl of DEVD-pNA (Asp-Glu-Val-Asp-p-nitroanilide) was added, incubated at 37°C for 4 h. The activated Caspase-3 levels were determined with amicrotiter plate reader, which can detect the absorbance of 400- or 405-nm. A negative control (sample with 50 μl PBS) and a positive control (sample treated with 10 μM H_2_O_2_ for 1 hour at 37°C) were used in all experiments.

### Determination of Nitric Oxide (NO) and NO Synthase (NOS) in Seminal Plasma

Total NO was measured spectrophotometrically as the manufacturer’s instructions using a commercially available kit (Jiancheng, Nanjing, Jiangsu, China). The kit involves the enzymatic conversion of nitrate to nitrite by the enzyme nitrate reductase, followed by colorimetric detection of nitrite. NO is synthesized from L-arginine in a reaction catalyzed by nitric oxide synthase (NOS), and then NO combines with a nucleophilic substrate to produce a chromophore that absorbs light at 530 nm. The assay for NOS is based on the quantitative conversion of oxyhemoglobin to methemoglobin by NO, which can be followed spectrophotometrically as a decrease in absorbance [29]. **Unit definitions:** One unit is defined as the amount of enzyme that will produce 1.0 nmol of NO per min at 37°C in 1.0ml seminal plasma. **Calculation formula:**

$$\begin{array}{l} \text{Total}-\text{NOS }\left(\text{U}/\text{ml}\right)=\frac{\text{OD tNOS }–\text{ OD Blank}}{\text{nanomole extinction coefficient of chromophore at }530\text{nm}}\;\times \text{ Df }\times \frac{1}{\text{nanomole extinctlight path pathreactiontim}} \end{array}$$

$$\text{Df}=\frac{\text{total volume of the reaction solution}\left(\text{ml}\right)}{\text{volumeof the sample used}\left(\text{ml}\right)}$$

### Measurement of the Concentration of Macrophage Migration Inhibitory Factor (MIF) in Seminal Plasma

The concentration of MIF in seminal plasma was assessed by using a specific human enzyme-linked immunosorbent assay (ELISA) kit (Jiancheng, Nanjing, China). Seminal samples were centrifuged for 10 min at 300 × g and then filtered with a 0.22 μm sterile Milex filter (Millipore, Billerica, MA). Forty μl of seminal samples, 10 μl of MIF-antibody and 50 μl of streptavidin-HRP were added into each test well. The plate was sealed with sealing memberance, and incubated for 60 min at 37°C. After removed the memberance carefully, the plate was washed five times. The chromogen solution (A) 50 μl and the chromogen solution (B) 50 μl were added into each well. To stop the reaction, 50 μl of stop solution were added into each well. Samples, MIF-antibody and Stretaptavidin-HRP were not added into the blank well, only chromogen solution (A) and chromogen solution (B) were added. Fifty μl of standard solution, 50 μl of streptavidin-HRP, chromogen solution (a) and chromogen solution (B) were added into each standard well, samples and MIF-antibody were not added. Take blank well as zero, the optical densit (OD) was measured under 450 nm wavelength within 10 min after adding the stop solution. According to standards’ concentration and the corresponding OD values, the standard curve linear regression equation was calculated out, and then the sample’s MIF concentration was calculated to base on the OD values of the sample.

### Statistical Analysis

All data were analyzed by one-way analysis of variance (ANOVA) using the SPSS 13.0 package software (SPSS Inc, Chicago, IL, USA). Differences between groups were verified by one way ANOVA. All the data were presented as mean ± SD. For results of HOS and HOS/AB, Paired-Samples *t* test was used. Two-tailed Pearson correlation test was used to assess the correlations between groups. A significant statistical difference was accepted when the *P* value was <0.05.

## Results

### Changes in Semen Parameters before, during and after SHS

**Fig 3:**
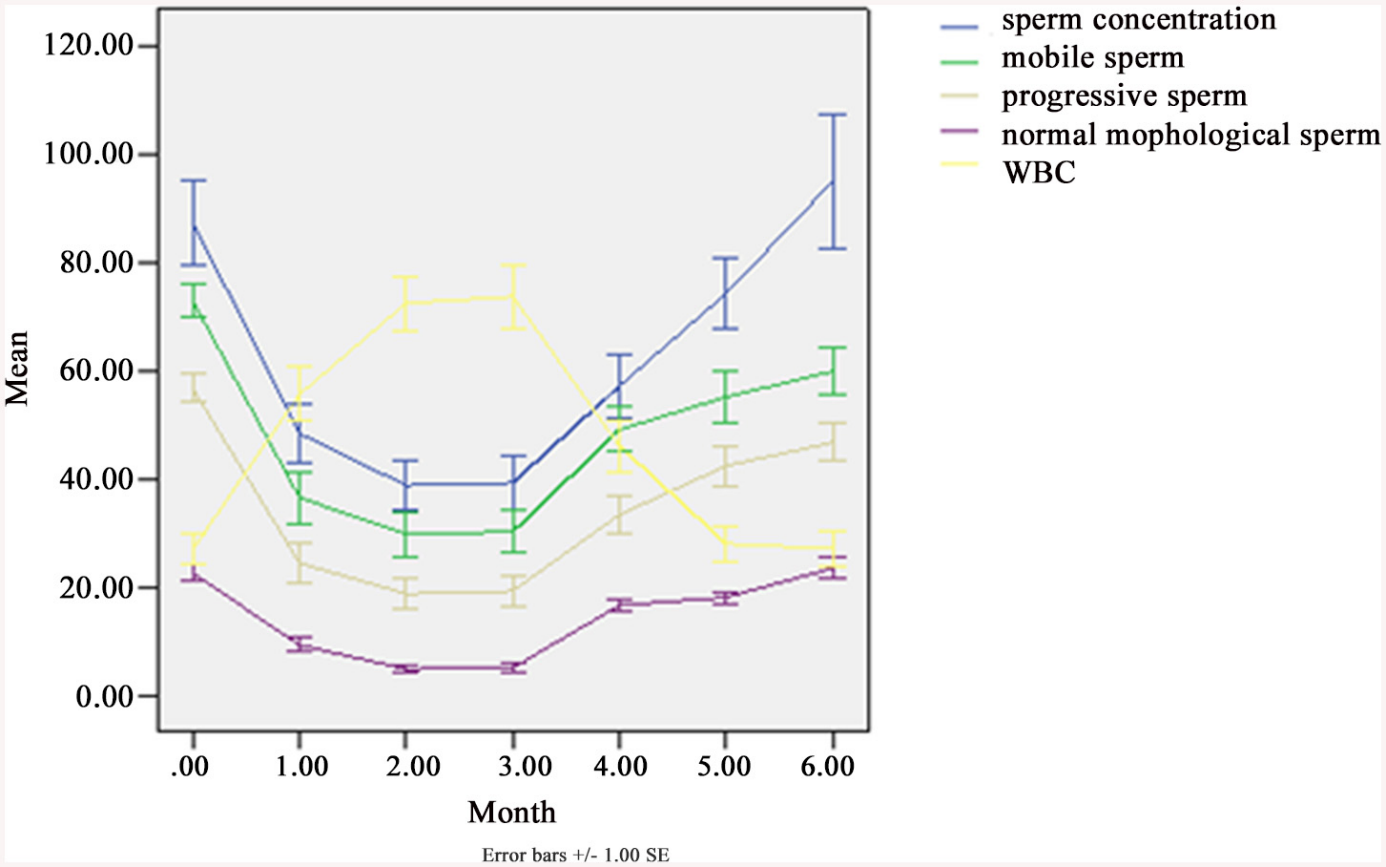
Sperm concentration, progressive motility (grade “a” + “b”), motile sperm, morphologically normal sperm and white blood cells (WBC) in semen, before, during and after SHS in 25 subjects. Concentration (× 10^6^/ml), Motile sperm = grade (“a” + “b” + “c”)%, Progressive = grade (“a” + “b”)%, Normal morphological sperm (%), WBC (× 10^6^/ml). From SPSS 13.0. Mean ± SEM.

Table 1 shows the changes in the sperm concentration, progressive motility (grade “a” + “b”), morphologically normal sperm and white blood cells (WBC) in semen, before, during and after SHS in 25 subjects. Before the SHS experiment, the range of sperm concentration was 24.27–167.53 × 10^6^/ml, the progressive motility rate (a + b) was 30%-84%, and the rate of the normal morphological sperm was 13%-45%. One month after SHS, the parameters were significantly altered: sperm concentration less than15 ×10^6^/ml in 6 subjects, and one dropped to 0; the rate of sperm progressive motility (a + b %) less than 20% in 7 subjects, the rate of morphologically normal sperm less than 5% in 9 subjects. Significant differences were observed in parameters of sperm concentration, motility, normal morphology and WBC between the pre-SHS group and the group during the SHS 1, 2 and 3 months (*P*<0.001). There was no significant difference in the semen volume between the SHS 1- and 2-month group and the pre-SHS group (*P*>0.05). The low semen volume observed in the pre-SHS group and the 3-moth SHS group (*P* = 0.018). After three months of recovery (stopped SHS), the semen parameters gradually returned to normal levels. Fig 3 shows sperm concentration, progressive motility (grade “a” + “b”), motile sperm, morphologically normal sperm and white blood cells (WBC) in semen, before, during and after SHS in 25 subjects.

**Table 1:** Analysis of variances of sperm density, progressive motility, morphology and WBC before, during and after SHS in 25 men using One Way ANOVA.

Variables	Pre SHS (n = 25)	SHS 1M (n = 25)	SHS 2M (n = 25)	SHS 3M (n = 25)	After SHS 1M (n = 25)	After SHS 2M (n = 22)	After SHS 3M (n = 20)	F value	*P* value
**Semen volume (ml)**	2.77 ± 0.56	2.65 ± 0.78	2.52 ± 0.59	2.30 ± 0.58	2.68 ± 0.72	2.92± 0.90	2.80 ± 0.67	2.030	0.065
**Sperm concentration (×10** ^**6**^ **/ml)**	87.41±39.11	48.35±26.99	38.77±23.39	39.26±25.44	51.14±29.69	74.81±33.92	91.07 ±49.36	10.429	0.000
**Progressive motility (a+b %)**	56.6 ± 13.0	24.4 ± 19.9	18.8 ± 14.8	19.3 ± 14.0	33.4 ± 17.1	45.1 ± 18.2	43.1 ± 16.3	20.072	0.000
**Normal morphology (%)**	22. 6± 7.7	9.3 ± 6.7	4.9 ± 3.8	5.1 ± 4.7	16.6 ± 5.6	18.5 ± 5.7	22.4 ± 8.3	37.070	0.000
**WBC in semen (×10** ^**6**^ **/ml)**	0.27 ± 0.15	0.56 ± 0.25	0.72 ± 0.25	0.74 ± 0.29	0.46 ± 0.24	0.28 ± 0.17	0.27 ± 0.13	20.826	0.000

### Results of Aniline Blue (AB) Staining, HOS (HOS/AB) and TUNEL Assay

The average percentages of AB1, AB2, AB3 and AB4 sperm from the HOS/AB test, unstained and stained sperm with AB and swollen and unswollen sperm with HOS for 25 subjects are shown in Table 2. There were no significant decreases between the sum percentages of AB1 plus AB2 and the percentage of unstained (normal) sperm with AB staining alone (*P*> 0.05), and between the percentages of AB3 and AB4 (abnormal) and the percentage of stained (abnormal) sperm with AB staining alone (*P*> 0.05), and between the percentages of AB3 and AB1 and the percentage of swollen sperm with HOS test. The sum of percentages of AB2, AB3 and AB4 sperm with HOS/AB was higher than that of stained sperm with AB alone (*P*< 0.05) and unswollen sperm (abnormal) with HOS test (*P*< 0.05).

**Table 2:** Patterns of the AB, HOS and HOS/AB test in 25 subjects before SHS.

Unstained (normal)	83.1±5.3 ^a^	Swollen	83.8±4.2 ^c^	AB1 (normal)	79.1±5.8
				AB2 (abnormal)	4.3±1.3
				AB3 (abnormal)	4.2±1.4
Stained (abnormal)	16.9±5.2 ^b^	Unswollen (abnormal)	16.2±4.2^b^	AB4 (abnormal)	12.4±4.9

Note: HOS/AB was observed for the same sperm. AB was only aniline blue for spermatozoa staining.

^a^ *P*<0.05, Paired-Samples *t* test, vs AB1;

^b^ *P*<0.01, Paired-Samples *t* test, vs AB4;

^c^ *P*<0.01, Paired-Samples *t* test, vs AB1.

### Results of Abnormal Sperm DNA Fragmentation, Chromatin Condensation, sperm Membrane Integrity and Caspase-3 Activity, and Levels of NO, NOS and MIF in Seminal Plasma

The percentage of sperm DNA fragmentation with TUNEL assay, abnormal sperm chromatin condensation with HOS/AB, normal sperm membrane and vitality with HOS, as well as Caspase-3 activity, and levels of NO, NOS and MIF in seminal plasma were compared before, during and after the use of SHS in 25 subjects (Table 3).

**Table 3:** Compared analysis of sperm DNA fragmentation, sperm chromatin condensation, the hypoosmotic swelling, level of NO, NOS, MIF as well as Caspase-3 activity before, during and after SHS in 25 men using One Way ANOVA.

Variables	Pre SHS (n = 25)	SHS 1M (n = 25)	SHS 2M (n = 25)	SHS 3M (n = 25)	After SHS 1M (n = 25)	After SHS 2M (n = 22)	After SHS 3M (n = 20)	F value	*P* value
**TUNEL assay abnormal sperm (%)**	11.7 ± 2.3	60.9 ± 28.1	70.0 ± 24.8	70.4 ± 25.1	19.8 ± 7.3	14.8 ± 4.0	12.4 ± 3.8	60.747	0.000
**HOS/AB abnormal sperm (%)**	20.9 ± 5.8	75.1 ± 23.7	80.2 ± 23.2	82.6 ± 20.6	29.4 ± 10.9	21.9 ± 6.2	21.1 ± 6.1	85.073	0.000
**HOS normal sperm (%)**	83.8 ± 4.2	33.1 ± 20.2	27.0 ± 14.9	27.6 ± 14.0	58.5 ± 8.2	80.7 ± 4.4	81.2 ± 6.0	115.837	0.000
**Level of NO (μmol/L)**	26.0 ± 11.2	38.7 ± 17.0	53.9 ± 21.6	59.8 ± 20.4	39.9 ± 14.4	30.0 ± 8.7	26.0 ± 13.4	16.511	0.000
**Level of NOS (U/ml)**	2.49 ± 1.65	3.96 ± 1.86	5.56 ± 2.57	6.32 ± 2.75	3.17 ± 1.82	2.41 ± 1.22	2.20 ± 1.31	16.168	0.000
**Level of MIF (ng/ml)**	380.8 ± 139.6	516.3 ± 154.6	557.6 ± 161.4	633.1± 204.4	451.4 ± 166.3	420.0 ± 149.4	420.5 ± 125.6	8.197	0.000
**Caspase-3 activity (U/10** ^**6**^ **sperms)**	1.81 ± 0.88	3.07 ± 1.42	3.63 ±1.05	3.54 ± 0.86	2.29 ± 0.73	2.41 ± 1.23	1.90 ± 1.03	12.291	0.000

**Fig 4:**
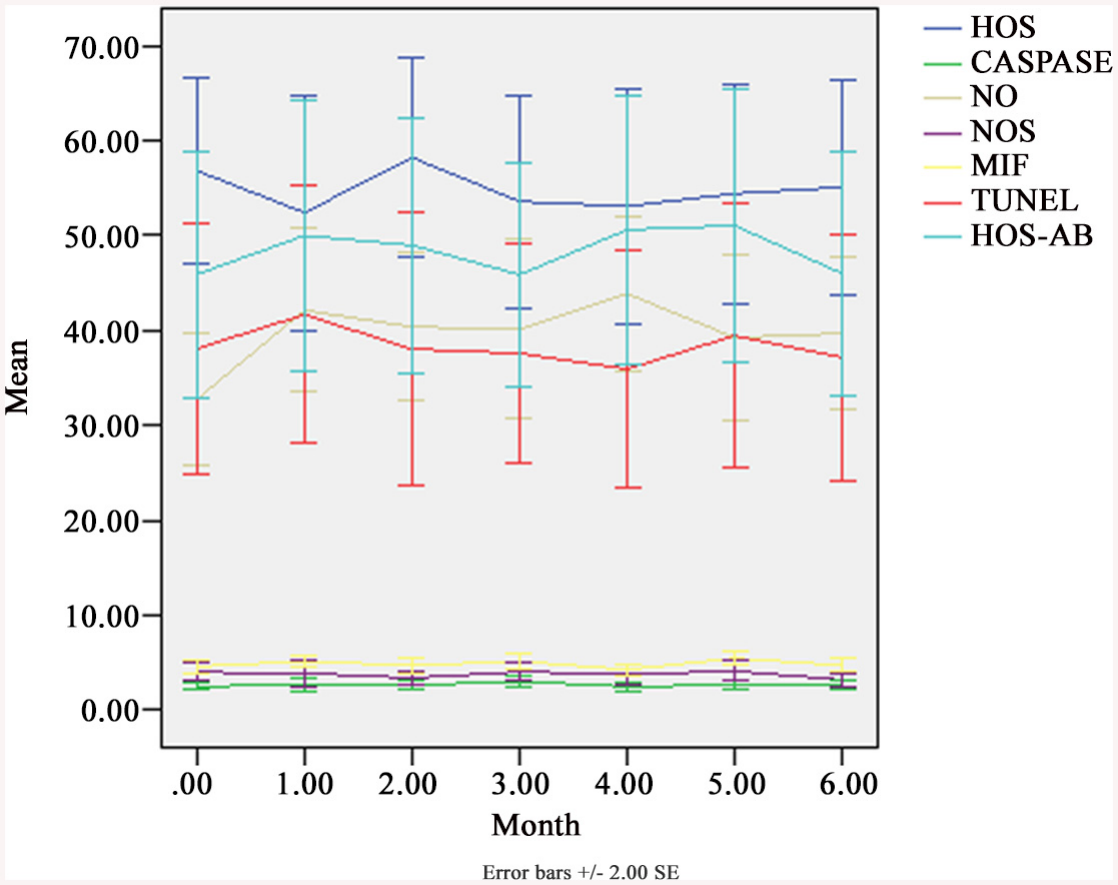
The percentage of sperm DNA fragmentation (%) with TUNEL assay, abnormal sperm chromatin condensation (%) with HOS/AB, normal sperm membrane and vitality (%) with HOS, as well as Caspase-3 activity (U/10^6^ sperms), and levels of NO (μmol/L), NOS (U/ml) and MIF (ng/ml) in seminal plasma were compared before, during and after the use of SHS in 25 subjects. From SPSS 13.0. Mean ± SEM.

Statistically significant differences of sperm DNA fragmentation, normal sperm membrane and vitality, Caspase-3 activity, and levels of NO, NOS and MIF were observed between the groups before SHS and the groups of during SHS 1, 2 and 3 months (*P*<0.001). The prevalence of abnormal sperm DNA, abnormal sperm chromatin condensation, normal sperm membrane and vitality, and Caspase-3 activity did not show any statistically significant difference between the groups before SHS and after SHS 3 months (*P*>0.05). Fig 4 shows the percentage of sperm DNA fragmentation with TUNEL assay, abnormal sperm chromatin condensation with HOS/AB, normal sperm membrane and vitality with HOS, as well as Caspase-3 activity, and levels of NO, NOS and MIF in seminal plasma were compared before, during and after the use of SHS in 25 subjects.

### Correlation among Sperm Parameters of the Conventional Semen Analysis, Sperm DNA Fragmentation, Abnormal Sperm Chromatin Condensation, Normal Sperm Membrane and Vitality, Caspase-3 Activity, and the Level of NO, NOS and MIF

A total of 167 semen samples were collected in 25 subjects before, during 1, 2 and 3 months, and after 1, 2 and 3 months of SHS. Three samples in the group with 2 months SHS treatment and five samples in the group with 3 months SHS treatment were not collected. Sperm concentration, motile sperm, progressive motility (grade “a” + “b”), normal morphology, and normal sperm by HOS test were negatively correlated with the percentage of abnormal sperm by using the test of TUNEL and HOS/AB, the levels of NO, NOS, Caspase-3 activity and MIF (*P*≤ 0.001). WBC in semen and Caspase-3 activity were negatively correlated with the percentage of normal sperm by using the HOS test (*P*≤ 0.001). Abnormal sperm with HOS/AB and WBC in semen showed a positively significant correlation with the level of NO, NOS and MIF, and Caspase-3 activity (*P*≤ 0.001) (Table 4).

**Table 4:** Correlation among sperm parameters of the routine semen analysis, sperm DNA fragmentation, abnormal sperm chromatin condensation, normal sperm membrane and vitality, and Caspase-3 activity, and levels of NO, NOS and MIF in seminal plasma (n = 167).

Variables	Sperm Concen-tration (×10^6^/ ml)	Progressi-ve motility	Normal mopholo-gy (%)	Abnorm-al HOS/AB (%)	Nor-mal HOS (%)	Caspas-e 3 activity	Level of NO (μmol/L)	Level of NOS (U/ml)	WBC (×10^6^/ml)	Level of MIF (ng/ml)
**Concentrat-ion (×10** ^**6**^ **/ml)**	*r* = 1									
**Progressive motility (a+b %)**	*r* = .459*	*r* = 1								
**Normal mophology (%)**	*r* = .540*	*r* = .701*	*r* = 1							
**Abnormal HOS/AB (%)**	*r* = .502*	*r* = -.633*	*r* = -.732*	*r* = 1						
**Normal HOS (%)**	*r* = .558*	*r* = .724*	*r* = .747*	*r* = -.864*	*r* = 1					
**Caspase-3 activity**	*r* = -.296*	*r* = -.292*	*r* = -.396*	*r* = .452*	*r* = -.455*	*r* = 1				
**Level of NO (μmol/L)**	*r* = -.228*	*r* = -.263*	*r* = -.390*	*r* = .469*	*r* = -.470*	*r* = .416*	*r* = 1			
**Level of NOS (U/ml)**	*r* = -.327*	*r* = -.395*	*r* = -.461*	*r* = .504*	*r* = -.520*	*r* = .584*	*r* = .416*	*r* = 1		
**WBC (×10** ^**6**^ **/ml)**	*r* = -.335*	*r* = -.385*	*r* = -.524*	*r* = .584*	*r* = -.603*	*r* = .410*	*r* = .428*	*r* = .495*	*r* = 1	
**Level of MIF (ng/ml)**	*r* = -.298*	*r* = -.395*	*r* = -.491*	*r* = .404*	*r* = -.447*	*r* = .291*	*r* = .267*	*r* = .405*	*r* = .665*	*r* = 1
**TUNEL assay abnormal sperm (%)**	*r* = -.516*	*r* = -.632*	*r* = -.632*	*r* = .901*	*r* = -.842*	*r* = -.433*	*r* = .411*	*r* = .486*	*r* = .579*	*r* = .409*

Pearson Correlation. *r*, correlation coefficient.

* Correlation is significant, *P* ≤ 0.001 (2-tailed).

## Discussion

Previous researches revealed a negative correlation between scrotal temperature and spermatogenesis has been demonstrated [30–32]. Local testicular heat treatment with 43°C water induced reversible oligospermia or azoospermia in rodents and monkeys with increased germ cell apoptosis [31–36], and when the heat treatment stopped after 30 days, the sperm quantity could recover to the original level. The heat treatment of the scrotum was mainly through the impact on the metabolism and apoptosis of spermatogenic cells to decline the fertility of many kinds of animal models; moreover, this impact was temporary and reversible [32–40].

In this study, we designed the SHSD with electric warming which was attached to the underpants for men, the temperature was 40–43°C and heating time was 40 min. After SHS 1 month, the parameters of sperm concentration, motile sperm and progressive motility (grade “a” + “b”), normal morphologic sperm were significantly decreased compared with that before SHS, and the WBC in semen was significantly increased. Moreover, the semen volume significantly decreased in samples from the group of SHS treated 3 months. After three months of recovery, the semen parameters gradually returned to the level before SHS treatment. These suggest that the scrotal heat stress in human for a short period of time may be affected to semen quality and may be a reversible process.

The testicular hyperthermia could cause a rapid and transient suppression of spermatogenesis and sperm DNA damage. In man, raised scrotal temperature can result in a negative correlation between high scrotal temperature and sperm output with sperm concentration being decreased 40% per 1°C increment of median day time scrotal temperature in a study of 99 men [41]. The heat stress not only directly induced the apoptosis of spermatogenic cells, but also impacted the cell division of the Sertoli cells to promote the apoptosis of spermatogenic cells [42–45]. In present study, when the SHS treatment was carried out 1, 2 and 3 months, the rate of the hypotonic swelling spermatozoa (the normal rate of HOS) were significantly declined, and the rate of the DNA-damaged sperm and abnormal chromatin condensation sperm as well as Caspase-3 activity also markedly increased than that before SHS (*P*<0.001). Three months after SHS treatment, the above-mentioned indicators were restored to the level before the SHS experiment. It prompts that the spermatogenesis might be a transient damage, when the SHS experiment stopped for a period of time, the spermatogenesis will recover. Wang et al [46] reported that after heat stress treatment, Caspase-3, -8, -9 enzyme activities in newt testis were significantly elevated after heat shock (40°C 2 h). When eight-week-old mice exposed to a single scrotal heat treatment (42°C for 25 min), the testes displayed severe damage, with multinucleated giant cells, nuclear condensation and germ cell loss in the seminiferous epithelium, and the number of cleaved Caspase-3-positive germ cells per tubule was dramatically increased [2,42].

In present study, we studied the sperm DNA fragmentation and sperm chromatin condensation using TUNEL and aniline blue (AB) assays. Sperm membrane integrity and vitality were used by HOS. We observed that sperm concentration, motility and normal morphology were negatively correlated with the percentage of abnormal sperm by using the test of TUNEL and HOS/AB, and with the level of seminal NO, NOS and MIF. Seminal NO, NOS, MIF, WBC and Caspase-3 activity were negatively correlated with the percentage of normal sperm by using the HOS test. Abnormal sperm with HOS/AB showed a negatively significant correlation with normal sperm by HOS test, and WBC showed a positively significant correlation with Caspase-3 activity and NO, NOS and MIF. The high correlation (correlation coefficient: 0.824–0.901) was detected between the HOS test, TUNEL assay and HOS/AB test. The normal spermatozoa in HOS test and HOS/AB test were membrane integrity and vitality, DNA integrity and chromatin not damaged. If the test only performed by one of the tests such as HOS or AB, some damaged spermatozoa may be as the normal sperm. The AB staining specifies sperm residual histones and indicates anomalies in sperm chromatin condensation. In earlier studies an association was reported between sperm developmental arrest, as demonstrated by aniline blue staining of persistent histones, and the number of chromosomal aberrations in semen samples [47–50]. These data lead us to a more detailed study regarding the relationship between sperm nuclear maturity and sperm membrane integrity and vitality as we pursued aniline blue staining and HOS within the same spermatozoon.

Nitric oxide (NO) is a free radical produced by most cells including the human male and female reproductive tracts and a well-known oxidative stress agent that directly inhibits mitochondrial respiration and the synthesis of DNA. Previous studies showed that human mature spermatozoa synthesize nitric oxide (NO) by the NO synthase (NOS) which exists as three isoforms—neural NOS (nNOS), endothelial NOS (eNOS), and inducible NOS (iNOS)–and localize in sperm head and midpiece [51, 52]. Increased NO synthesis through up-regulation of iNOS has been implicated in cellular injury and apoptosis in various cell systems [53–56]. MIF is a well-known proinflammatory mediator and may have important functions in human reproduction and prostatic physiology. Interestingly, it also suggested that there is a negative correlation between the amount of sperm-associated MIF and the percentage of motility in different semen samples [57]. Although several studies have proposed the co-relationship between infertility and semen NO and MIF concentrations, no study on the effects of scrotal heat stress (SHS) has been reported.

In summary, our current study evaluated the changes in semen parameters, sperm DNA integrity, sperm chromatin condensation, and sperm Caspase-3, seminal levels of NO, NOS, MIF in men after SHS. Our data showed that in men with the treatment of SHS, the semen quality was significantly decreased and sperm membrane, DNA and sperm chromatin condensation were damaged. These may be contributed to high levels of NO, NOS, MIF and Caspase-3 in seminal plasmas.

## Supporting Information

S1 Data (SAV)
